# Book Reviews

**Published:** 1819-08

**Authors:** 


### CRITICAL ANALYSIS

OF

### RECENT PUBLICATIONS, IN THE DIFFERENT BRANCHES OF MEDICINE AND SURGERY;

### SELECT MEMOIRS, AND HISTORIES OF CASES;

### In the Literature of Foreign Nations.

Πατρίδες ἄρα
Ανδράσιν, ὧ πατρὰς ἄνδρες, ἀγαλλόμεθα.

*The Medical and Surgical Register; consisting chiefly of Cases in the New-York Hospital.* By JOHN WATTS, jun. M.D. VALENTINE MOTT, M.D. and ALEXANDER STEVENS, M.D. 8vo. pp. 163. Collins and Co. New-York. 1818.

THE most important part of this volume is that which comprises a *history of the application of a ligature to the arteria innominata in a man*, by Dr. V. MOTT. We remark in the first instance, in order to show at once the object of our strictures on this history, that it is by no means clear, or indeed probable, that the disease for which the ligature was employed was an *aneurism of the subclavian artery*, as supposed by Dr. Mott; the gratification we have experienced from viewing in this case an effort of the art of surgery that may hereafter be employed with the most beneficial effects, is, therefore, accompanied with sensations of painful regret, from a fear that this knowledge has been obtained by the sacrifice of the life of a human being.

Having made this statement, we feel obliged to enter much into detail in our account of this case: we shall therefore, in the first instance, wholly transcribe the history of it previously to the operation.

"Michael Bateman, aged 57 years, was born in Salem, Massachusetts, and by occupation a seaman. He was admitted into the New-York Hospital, on the 1st of March, 1818, for a catarrhal affection, having at the same time his right arm and shoulder much swollen. At the time of his admission, the catarrh being thought the most considerable disease of the two, he was received as a medical patient, and placed under the care of the physician then in attendance. During the three first weeks of his residence in the house, the catarrh had greatly yielded to the remedies prescribed. The inflammation, which had produced an enlargement of the whole superior extremity, extending itself to the muscles of the neck on the right side, was also gradually subsiding.

"A tumefaction, however, situated above and posterior to the clavicle, at first involved in the general swelling, and not to be distinguished from it, began to show itself. This resisted the remedies which were effectual in relieving the other, and became more distinct and circumscribed as the latter subsided; at length assuming the form of an irregular tumor.

"The history which he gave of the case is as follows: He said, about a week before he entered the hospital, while at work on shipboard, his feet accidentally slipped from under him, and he fell upon his right arm, shoulder, and the back part of his head; that he felt but little inconvenience from the fall, and after a short time returned to his duty. Two days subsequent to this, however, he felt pain in the shoulder, and the succeeding night was unable to lie upon it in bed. The whole arm and shoulder then began to swell, and became so painful, that he was unable any longer to perform his duty as a seaman. The ship having arrived in New-York, he was admitted into the hospital.

"For some time after the general swelling had subsided, leaving the tumor distinct and circumscribed, no circumstance occurred which gave rise to a suspicion of its being aneurismal. The enlargement was thought to be a common indolent tumor, and was repeatedly blistered, with a view to discuss it. The tumor gradually diminished under this treatment, though a considerable time elapsed before any very striking change took place.

"At length a faint and obscure pulsation was perceived: still it was a matter of doubt whether the tumor was aneurismal, or whether the pulsatory motion was communicated to it by the subclavian artery, immediately over which it was situated. From its firm, unyielding nature, upon pressure, the latter was considered as the most probable, and the blisters were continued as before. During the whole of this time the patient had worn his arm in a sling, the motions of it being very limited, and always attended with pain.

"The patient remained in this state for several days, without any marked change either in his feelings or in the appearance of the tumor.

"On the 3d of May, at six o'clock in the afternoon, the patient complained that he 'felt something give way in the tumor;' that his shoulder was very painful, and that he was able to raise it only a few inches from his side. The tumor at this time suddenly increased about one-third, and a pulsation was distinctly perceptible. Its most prominent part was below the clavicle, at which place the pulsation was most distinct. The portion above the clavicle was also much enlarged; it still however had its usual firmness, except in one point near its centre.

"*May 4th.*—The tumor is evidently increased, that portion of it more particularly which is below the clavicle; it is not as firm and resisting as it has been. Pulsation is not so distinct as yesterday, but appears to be more diffused.

"He was this day transferred to the surgical side of the house, and became my patient. The cough having become comparatively slight, the tumor appeared to be the most urgent disease, and, in my opinion, to call for prompt attention. The arm is now perfectly useless, and any motion at the shoulder-joint gives him severe pain. The patient is naturally of a spare habit; and, from the nature of his disease, and the confinement to which he has been subjected, has become much reduced in strength.

"*May 5th and 6th.*—The tumor is still progressing, and the pain in the shoulder is also more severe. During the three last days his medicines have been discontinued, except that he is allowed to rub the parts about the clavicle with volatile liniment.

"On the 7th, I directed a consultation of my colleagues to be called, consisting of Drs. Post, Kissam, and Stevens. I now stated to them that I wished to perform an operation which would enable me to pass a ligature around the subclavian artery, before it passes through the scaleni muscles; or the arteria innominata, if the size of the tumor should prevent the accomplishment of the former. This I was permitted to do, provided the patient should assent, after a candid and fair representation was made to him of the probable termination of his disease; and that the operation, though uncertain, gave him some chance, and, as we thought, the only one, of his life.

"Dr. Post, at my request, communicated with him privately on this subject; and, after a full explanation of the nature of the case, my patient requested to have any operation performed which promised him a chance for his life, saying, that in his present state he was truly wretched.

"*May 8th, 9th, and 10th.*—The tumor is acknowledged by all to be increasing, and it is thought proper not to defer the operation any longer: I therefore requested that preparation be made for performing it to-morrow.

"It is difficult to give an idea of the size of a tumor so irregular in its form, and so peculiarly situated. A thread passed over it, from the lower part of that portion of it which is below the clavicle, extending upward obliquely across the clavicle toward the back of the neck, will measure five and a quarter inches. Another crossing this at right angles one inch above the clavicle, will measure four inches; two and a half inches of the thread are on the sternal side of the former, and one and a half on the acromial. It rises fully an inch above the clavicle; which, added to the depression below the clavicle on the opposite shoulder, will make the size of the swelling above the natural surface about two inches."

When we consider, that this tumor appeared with, or immediately subsequent to, the inflammation of the arm, shoulder, and neck; that it, for some time after it was first discovered, *diminished in volume, and was without pulsation;* that it was of an irregular form, and of a "firm, unyielding nature, on pressure;" that, when pulsation became evident, this pulsation was only in the centre of it, the rest of the tumor being of "its usual firmness;" that, at length the pulsation suddenly became less distinct and more diffused, the tumor increasing one-third in size, but retaining its firmness, except in one point near its centre; and lastly, the great bulk it acquired in so short a period; we are utterly at a loss to conceive how Dr. Mott could have imagined it to be an arterial aneurism. There is not a phenomenon or symptom amongst those he has related, that, in our opinion, should have made him alter his original idea of the nature of the disease: the whole of the latter series of symptoms only indicated that suppuration had taken place in a tumor situated upon the subclavian artery. The "something that give way in the tumor," noticed in the report of the 3d of

May, was probably the bursting of the tunics of an abscess: this is indicated by the whole of the report of that and the following day.

But, as the reader must be anxious for the remainder of the history of this case, we proceed in its relation : our further remarks will then follow.

We need not describe particularly the preliminary steps of the operation, since every anatomist must be acquainted with those which are proper on such an occasion: it may suffice to observe, that an incision was first made from just above the right clavicle and upper end of the sternum, extending over the trachea, three inches in length ; then another incision, about the same length, extended from the termination of the first along the inner edge of the sterno-cleido-mastoid muscle; the integuments were then dissected from the platisma myoides, which, with the sternal and part of the clavicular portion of the sterno-cleido-mastoid muscle, the sterno-hyoid and thyroid, were separated from their lower attachments, and reflected upwards over the tumor. The sheath containing the carotid artery, par vagum, and internal jugular vein, was thus exposed a little above the sternum; the artery was then exposed, and the vein and nerve separated from it; and, tracing it from this point, the subclavian artery was readily laid bare, about half an inch from its origin.

" On arriving at the subclavian artery, it appeared to be considerably larger than common, and of an unhealthy colour; and, when I exposed it to the extent of about half an inch from its origin, which was all that the tumor would permit, to ascertain this circumstance more satisfactorily, my friends concurred with me in opinion, that it would be highly injudicious to pass a ligature around it. The close contiguity of the tumor would of itself have been a sufficient objection to the application of the ligature in this situation, independent of the apparently altered state of the artery. Art, in this case, could not anticipate anything like the institution of the healthy process of adhesive inflammation in an artery in the immediate vicinity of so much disease.

" With this appearance of disease in the subclavian artery, it only remained for me either to pass the ligature around the arteria innominata, or abandon my patient. Although I very well knew that this artery had never been taken up for any condition of aneurisms, or ever performed as a surgical operation, yet, with the approbation of my friends, and reposing great confidence in the resources of the system, when aided by the noblest efforts of scientific surgery, I resolved upon the operation.

" The bifurcation of the innominata being now in view, it only remained to prosecute the dissection a little lower behind the sternum. This was done mostly with the round-edged knife, taking care to keep directly over and along the upper surface of the artery. After fairly denuding the artery upon its upper surface, I very cautiously, with the handle of a scalpel, separated the cellular substance from the sides of it, so as to avoid wounding the pleura. A round silken ligature was now readily passed around it, and the artery was tied about half an inch below the bifurcation. The recurrent and phrenic nerves were not disturbed in this part of the operation."

No change whatever took place in the countenance of the patient on the tying of the ligature, and

"Dr. Post now asked him, if he felt any unpleasant sensation about his head, breast, or arm, or felt any way different from common? to which he replied, that he did not.

"Immediately after the ligature was drawn tight, the tumor was reduced in size about one-third, and the course of the clavicle could be distinctly felt.

"Ten minutes after the operation, the pulse is regular, and not the least variation can be perceived: it beats sixty-nine strokes in a minute; the patient says he is perfectly comfortable, and has no new or unnatural sensation, except a little stiffness of the muscles of the neck, which he thinks is owing to the position in which his head was placed during the operation. The temperature of the right arm is a little cooler than the left; his breathing has not been the least affected by the operation, but is perfectly free and natural.

"2 *o'clock* P. M.—Patient expresses a desire to eat, and is directed a little thin soup and bread; the temperature of both arms is very nearly the same; breathing perfectly natural; pulse as before.

"3 *o'clock* P.M.—There is still a trifling difference in the temperature of the two arms; ordered the right to be wrapped in cotton wadding; not the least unpleasant symptom has as yet made its appearance.

"6 *o'clock* P M.—Complains of a little pain in his head, not more on one side, however, than the other; describes it as a common head-ache. The pain of the shoulder and arm much less than before the operation: no difference can now be perceived in the temperature of the two arms; pulse a little accelerated, and perhaps a little full.

"9 P.M.—Patient complains of head-ache; skin is rather hotter than natural; pulse strong and full, and beats 75 in a minute; the carotid on the left side of the neck is observed to be much dilated, and in strong action; tongue moist and clean.

"$9\frac{1}{2}$ P.M.—Symptoms continuing the same, directed him to be bled from the left arm to ℥ xvj. After bleeding, the pulse fell seven beats, and was less full. Complains of some thirst; let him drink common tea.

"12 P.M.—Patient has slept a little; is free from pain; pulse full and less frequent, beats 60; skin moist, and of a natural temperature."

We have wholly transcribed the report of the first day, in order to gratify the desire of the reader to know the immediate consequences on the vital phenomena in general of so important an operation. Nothing remarkable occurred on the following day: the patient, however, lost eight ounces of blood, for the relief of a little head-ache that supervened. On the third day, it is said that "the size of the tumor is considerably diminished." We shall detail every observation relative to this point. We pass on to the report of the fifth day, since the little disturbance of the vital functions that ensued from the operation, is not of sufficient importance to be detailed: the slight degree of derangement noticed in them is the most remarkable circumstance.

The wound was first dressed on the fifth day. On removing a yest poultice, which had been applied because there had been a discharge of fetid matter, it was found that the "suppuration was considerable, and of a healthy appearance." On the sixth day, "the dressings were again removed, and the discharge seemed more considerable than at the former dressing." On the seventh day, "the suppuration was considerable and healthy." On the eighth, "the suppuration is more profuse, apparently healthy, though attended with considerable fœtor." On the ninth day, the discharge was less in quantity, and less fetid; "the tumor has diminished two-thirds, is soft, and less florid. The apex of the tumor is now below the clavicle." In the evening of the same day,

"When preparing to renew the dressings, an unexpected and an unaccountable hæmorrhage took place, which suddenly filled the cavity of the wound. The rapidity with which the blood flowed, and the size of the stream, gave rise to fearful apprehensions for the man's safety. Dry lint was immediately placed in the wound, and as much pressure made as the patient could conveniently bear, which quickly stopped it. After continuing the pressure for a short time, the lint was removed, when no hæmorrhage recurring, the usual dressings were repeated. The patient experienced no ill effects from the bleeding, nor did he seem to be much agitated."

On the eleventh day, it is stated that

"The wound is dressed daily at this hour (11 A.M.): its appearance is still very favourable, although there is still some fetor in the suppuration. The wound has contracted perhaps one-third; the tumor is also considerably diminished, and softer than before; pulsation in the right temporal and radial arteries, as before. The same dressings to be continued."

On the following day, a slough, "apparently a portion of the sheath of the vessels, came away;" as well as "the ligature which had been applied to an artery under the lower portion of the thyroid muscle." Within a few days after this, the suppuration became but very small in quantity. The large ligature came away on the fourteenth day. We now pass on to the report of the twenty-first day, which we give entirely.

"Dressed the wound three times again to-day: it is nearly closed at the bottom. The power of motion in the right arm continues to increase: he can now move it with as much facility as the left, though not to the same extent. His strength is daily improving, and the operation is considered by all to have been completely successful. Size of the tumor continues the same, no diminution of it having been perceived for the last week. The most prominent part of the tumor is yet below the clavicle; that above rises to about the height of the clavicle, which gives a little convexity to the place between the clavicle and trapezius muscle."

On the twenty-third,

"A few minutes before the hour of visiting to-day, a message was brought that the patient was bleeding from the wound. The dressings were immediately torn off, and dry lint crowded into the wound, and slight pressure applied for a few minutes, when the hæmorrhage ceased. The patient lost at this time perhaps about twenty-four ounces of blood, and was very much prostrated. Pulsation ceased in the radial artery of the left arm, and the countenance, gasping and convulsive throes of the patient, threatened immediate dissolution: all present apprehended the instant death of the patient. . . . . He says, that he felt weary of lying on his left side and back; that he had just turned on the right, which he had not done before since the operation, agreeable to my request. At the instant of turning over, something arrested his attention, which caused him to turn his head to the opposite side suddenly, and he felt the gush of blood from the wound."

He took a pint and a half of madeira wine in the ensuing twenty-four hours.

"*Twenty-fourth day*, 6 A.M.—Slept the greater part of the night, and feels comfortable; is still languid, and has no disposition to eat any thing; says he feels sick, and once last evening vomited after drinking some wine and water.

"Wound looks exceedingly pale, and the discharge is thin and fetid, for which the carbon and yest dressings were applied. He has vomited several times to-day, and has some considerable difficulty in swallowing, and complains of a soreness in the wound upon pressure.

"9 P.M.—Dressings removed; wound very pale; right arm of the natural temperature; feels occasionally a little numbness in the hand; has taken very little nourishment during the day; pulse natural as to frequency, but small and feeble. A few minutes after dressing the wound, information was brought that hæmorrhage had ensued; and, before it could be commanded, he probably lost four ounces of blood. For his restlessness and pain in the bones, he was ordered two grains of opium.

"*Twenty-fifth day.*—Has rested well during the night, and is perhaps a little better this morning. The repeated hæmorrhages have debilitated him exceedingly; and, from the irritable state of the stomach, he can only take a very little nourishment. In the morning he was directed the effervescing draught, to be repeated every two hours: this allayed the irritability of his stomach, and enabled him to take a little breakfast.

"His countenance has altered since the first bleeding surprisingly: his eyes are now heavy, and for the most part fixed; his cheeks are sunken, and an universal palor has spread itself over his countenance; and, from every appearance, a short time will terminate his existence. He has not vomited since early in the morning; is advised to take a little soup, and to drink freely of wine and water. Dressings were renewed at 3 o'clock P.M.; shortly after which the patient again bled, but not to exceed, however, an ounce.

"*Twenty-sixth day*, 6 A.M.—Patient has not rested well; is occasionally falling into little slumbers, but is awaked by the least motion. Pulse small and feeble; respiration somewhat laboured; appears to be sinking; seems disinclined to take any thing; legs and arms constantly in motion.

"6 P.M.—Is extremely low; respiration very much laboured; is not able to articulate. For the last three hours there has not been such continued throwing of the legs and arms about the bed: he lays in a state of insensibility; temperature of the two arms the same to the last."

Soon after this "there was another bleeding from the wound, by which he lost probably eight ounces of blood. During the whole time he did not manifest the least appearance of consciousness, nor was the least motion perceptible, except that necessary for respiration and circulation. The hæmorrhage was stopped with lint, after removing the former dressings. Respiration is now performed with the utmost difficulty, and the patient appears as if every respiration would be the last. He expired at half-past six in the afternoon. The temperature of the right arm after death, appeared by the touch to be the same as the left; it was as natural and uniform as other parts of the body."

At present we only remark, that the cause of the death of the patient is not clearly evident: the hæmorrhage in itself was not sufficient to account for it in a satisfactory manner. We transcribe wholly the account of the examination of the body after death.

"About eighteen hours after death, I opened his body. There was considerable emaciation, and the surface of the wound was of a dark-brown colour, and fetid; the wound was perhaps about one-third of its original size; it had been enlarged by the pressure of lint into it, and other means to arrest from time to time the hæmorrhage: the ulcer between his shoulders was ill-conditioned.

"For the purpose of examining the condition of the aorta, where the arteria innominata is given off, as also the origin of the latter vessel, as well as the state of the pleura at the part about which the ligature had been applied around the artery, the chest was opened in the following manner: After removing the integuments and muscles from the forepart of the chest, the sternum was carefully sawed through about an inch from its upper extremity, and raised by sawing through the ribs below the junction of the cartilages: this removed so much of the front part of the chest, as to facilitate and expose fully to view the subsequent steps of the dissection. By thus leaving the clavicles attached, every part connected with the ulcer and great vessels could be seen, and examined *in situ*.

"The arch of the aorta, and origin of the innominata, being fairly exposed, not a vestige of inflammation or its consequences could be discovered, either upon them, the lungs, or the pleura, at any part. An incision was next made longitudinally into the aorta, opposite the origin of the innominata; and, upon introducing a probe cautiously up the latter vessel, it was seen to pass into the cavity of the ulcer. The innominata was then laid open, with a pair of scissors, into the ulcer: the internal coat of this vessel was smooth and natural about its origin, but for half an inch below where the ligature had cut through the artery, it showed appearances of inflammation, and there was a coagulum adhering with considerable firmness to one of its sides; showing that nature had made an effort to plug up the extremity of so large a vessel, after the adhesion, which no doubt had been effected by the ligature, was swept away by the destructive process of ulceration.

The upper extremity of this vessel was considerably diminished in its diameter by the thickened state of its coats, occasioned by the surrounding inflammation. The innominata, about half an inch from the aorta, and a little to the left side, gave off an anomalous artery, large enough to admit a small size crow-quill.

"The ulcer at the bottom was more than twice the size of the wound in the neck; it extended laterally towards the trachea, and under the clavicle towards the tumor. The tripod of great vessels, consisting of the innominata, subclavian, and carotid arteries, to the extent of nearly an inch, was dissolved and carried away by the ulceration. The extremities of the two latter vessels were found also to open into the cavity of the ulcer. The upper surface of the pleura was very much thickened by the deposit of newly-organized matter, for the safety and protection of the cavity of the thorax. Indeed, instead of having increased the danger of penetrating this membrane, the adhesive inflammation which preceded the ulcerative, seemed, by the consolidation of cellular membrane, and the addition of new substance, to have more securely and effectually shielded it from danger.

"The internal surface of the carotid artery was lined with a coagulum of blood, more than twice the thickness of its coats, and extending above the division into internal and external, so as almost to give them a solid appearance, insomuch that a probe could barely be introduced. The subclavian artery, internally and externally to the disease, was pervious. The brachial and other arteries of the right arm were of their common diameter, and in every respect natural. The external thoracic or mammery arteries, as they went off from the subclavian, were larger than natural; the right internal mammary was pervious, and of the usual appearance. Upon opening into the tumor, which now gave (from its small size) no deformity to the shoulder, the clavicle was involved in it, and found carious, and entirely disunited about the middle. A number of lymphatic glands under the clavicles, and particularly the left, were considerably enlarged; and, when cut into, very soft, and evidently in a state of scrofulous suppuration. No other morbid appearances were observed."

What a vague and obscure account! Nothing satisfactory is said respecting those points on which every reader must have waited with anxiety for precise information. The author, from a sense of duty to his own reputation and to the dignity of the art which he professes, should have either shown that an aneurism of the subclavian artery had existed, or have candidly acknowledged that he had mistaken the nature of the case. We know not, indeed, how to interpret the account here given of the appearances after death: it has not the character of a cautious and deliberate avoidance of those points which should have formed the most important part of the anatomical investigation. We are disposed to consider, that, as Dr. Mott had himself no doubt of the existence of arterial aneurism, he had no care to make it appear evident to the public. Yet, when we consider that Dr. Mott has shown, on other occasions, that he possesses at least common accuracy of discernment, we are surprised that the history of the case during life, and the appearances on dissection, (as far as the obscurity of the account here given will permit of rational inductions from it,) did not make it evident to him that doubts respecting the existence of aneurism might, with the strictest propriety, be entertained; and that a more accurate history of the case would be required from him than that which he has here produced. We find, indeed, no well-marked symptom or sign of aneurism, but many circumstances which mutually contribute to show that the disease was a suppurating tumor, situated over the subclavian artery. We have no description of an aneurismal sac; but it is vaguely said, "the subclavian artery, internally and externally to the *disease*, was pervious." Place this observation in opposition with the following: "a number of lymphatic glands under the clavicles, and particularly under the left, were considerably enlarged, and, when cut into, very soft, and evidently in a state of scrofulous suppuration;" and consider attentively the whole history of the case, and we think there can be but one conclusion formed as to its nature.

The reduction of the size of the tumor "about one-third" on tying the ligature, is the only occurrence noticed in this history, indicative of arterial aneurism; and this may be very well explained by the subsidence of the artery on which it rested, and of the neighbouring parts, in consequence of the comparative emptiness of the vessels thus induced. We must not omit to remark, too, that not so much further diminution of its volume ensued, as should be expected were it an aneurism, particularly when we consider the destruction of the surrounding parts from ulceration that afterwards took place. On the third day after the operation, it is stated that "the size of the tumor is considerably diminished;" on the ninth day, the "tumor has diminished two-thirds, is soft, and *less florid;* on the eleventh, it is said to be "considerably diminished," without mentioning whether this is regarded in comparison with the last report, or with its original size; and after this, "no diminution of it had been perceived for the last week:" a few days afterwards, the patient died.

There is another point we cannot suffer to pass without some remarks: the propriety of applying the ligature to the innominata, rather than to the subclavian artery, is not determined. The reasons given for not placing the ligature on the latter vessel, is that it "appeared to be considerably larger than common, and of an unhealthy colour," are not satisfactory, particularly as regards the first of these two observations. The author says, there was but half an inch of it extraneous to the tumor; and that "the close contiguity of the tumor would of itself have been a sufficient objection to the application of the ligature in this situation, independent of the apparently altered state of the artery." This we consider by no means a fair or correct conclusion. The author again says, "the pathology of arteries has long since taught us, that ulcerative inflammation, and all its train of consequences, would have been the inevitable result. This was the fate of the only case in which a ligature has been applied to the artery in this situation." Dr. Mott's pathological argument is by no means well-founded; and his corollary, according to his own mode of reasoning, should have contra-indicated the operation altogether. It is the case related by Dr. Colles, of Dublin, to which Dr. Mott here alludes. But we must not permit ourselves to attribute much importance to Dr. Mott's physiological reasoning, after finding him say that "the brain, in no operation, has been deprived of so large a quantity of blood as in this, and yet it suffered no inconvenience: *from the effect of experiments, however, upon animals, I entertained no fear as to the consequences of my operation upon this organ.*" (p. 52.) It is strange that men will draw from experiments on brute animals conclusions they do not admit of, and neglect those inductions which may thence be rationally formed. The whole of the cerebrum may be removed from a dog without the immediate destruction of its life; he will indeed walk about after the loss of it: but no rational person can suppose that such an operation would be attended with similar results in a human being. The relation of the functions of the brain to those of the rest of the body, are widely different in the different classes of animals.

Returning to the state of the subclavian artery: the only observation made respecting it on dissection, is, that "internally and externally to the disease it was pervious;" except that it is stated, that "the tripod of great vessels, consisting of the innominata, subclavian, and carotid arteries, to the extent of nearly an inch, was dissolved and carried away by ulceration."

It appears that Dr. Mott applied the ligature to the artery without exposing the cavity of the thorax, which was one of the circumstances to be apprehended in the operation. This, we have ascertained, may be effected very well in dogs, by seizing the periods of expiration for making the necessary incisions in the exposure of the artery, and the tying of the ligature.

On concluding the remarks on this history which the nature of the serious duties we assume has obliged us to adduce, we repeat, that the gratification derived from the useful indications it furnishes, is bitterly alloyed by regret for the fate of the unfortunate patient, and disgust at the manner in which so important an event is recorded. We are also disposed to add, that the idea of the practicability of this operation, which Dr. Mott has "been in the habit of showing in his surgical lectures," seems to have preyed upon his mind, and led him to seize, in a hot and hasty manner, this opportunity for putting it to the test of experience. This remark would apply to the case, even were it proved to have been an arterial aneurism.

The next article in this volume is

*A Case of Injury by Lightning, successfully treated by copious Venesection.* By Alexander H. Stevens, M.D.

The very interesting nature of this case, and the results of the active and judicious treatment had recourse to in it by Dr. Stevens, induce us to give it in detail.

"I. Stones was brought into the New-York Hospital on the 29th April 1818, having been struck with lightning a week previous on board the ship Sea-Fox, Captain Fanning, off New-London. It appeared that his left hand grasped a rope, which he was drawing down; his left foot was probably at the same moment raised from the deck of the vessel. The electric fluid, in descending the rope, had passed diagonally across his body, from the left hand to the right foot, injuring in its passage the intermediate viscera, and a considerable breadth of external integuments. A reflected operation had also involved the left side of the neck and head. The whole surface of the affected skin was blistered, as if with hot water; and numerous points were scattered over it, resembling punctures with a fork or nail. The poor fellow exhibited no signs of life for several hours after the stroke. Nothing had been done for him before he was brought to the hospital. His skin was heated and dry, his breathing laborious, his pulse strong and frequent, his tongue white and dry, and his pupils dilated. Under these circumstances, I directed the vesicated parts to be covered with the ordinary lime-water liniment. Venesection and the antiphlogistic treatment, together with the compound powder ipecac. and antimonials, were accordingly directed. Notwithstanding the typhoid heat of the skin, dilated pupil, occasional delirium, and great nervous agitation, the amount of blood drawn within ten days was about 120 ounces: it constantly exhibited the buffy coat. The recovery of the patient, a long time doubtful, and protracted by great nervous debility, was at length completed; and, at the expiration of seven weeks, except a degree of weakness in the right knee, his health was perfectly re-established."

*Remarks on Cataract, and on the Position of the Patient in Operations on the Eye; with a Case successfully treated.* By Alexander Stevens, m.d.

The author's remarks convey no ideas either of novelty or importance. The position of the patient recommended is the recumbent one, "during an operation on the right eye, as far more convenient even to those who possess the happy faculty of dextrously using the left hand."

*Remarks on Ligatures for Arteries.* By Alex. H. Stevens, m.d.

In this paper, after having made some common-place remarks on the different sorts of ligatures for arteries that have been employed, the object of which is to show that the old and common one is the best, the author says, "Perhaps we may soon hear of an operation for aneurism without tying the artery; but only surrounding it loosely with a ligature, the irritation of which would, it is believed, cause an effusion of lymph about and within the vessel, and gradually obstruct the transmission of blood through it." A crude and ill-founded speculative notion, which we heard, some years since, advanced on this side the Atlantic by a lecturer on anatomy and surgery in one of the London schools; but which we suppose will not be put in practice at the hazard of a patient's welfare.

*A Case of Compound Fracture of the Os Femoris, which terminated favourably.* By Valentine Mott, m.d.

There is nothing remarkable in the history of this case.

### *A Case of Stricture in the Urethra, cured by an Operation.*

By ALEXANDER STEVENS, M.D.

In this case the patient had two strictures: one, two inches and a half from the orifice of the urethra, an inch in extent, "impenetrable to the finest probe, and almost as hard as bone;" another, about an inch further onward towards the bladder. The ordinary methods having been ineffectually employed, and the patient suffering great inconvenience; "the bladder remained constantly distended, and he was under the necessity of carrying a vessel to receive the urine which was constantly dribbling from the urethra;" an opening was made into the urethra, in the situation of the bulb: the lower stricture was then destroyed by caustic; and afterwards, because the man was impatient of the slowness of his cure, Dr. Stevens says, "I passed a probe into the meatus urinaris down to the stricture. The space between the end of the probe and that of a bougie passed upward, is more than three quarters of an inch. I laid open the urethra between them, cut away the hardened portions of it, passed a full-sized bougie into the meatus, past the strictures, and through the opening in perineo." The patient finally recovered.

### *Remarks on the supposed Effects of drinking Cold Water; illustrated by Cases which occurred during the Hot Weather of the Summer of* 1818: *submitted to the Medical Society of the County of New-York.* By JOHN WATTS, jun. M.D.

This paper contains an account of some very interesting cases of disease, the value of which is much increased by the judicious remarks of the author. We shall give the first series of observations in his own words:

"During the last two days of June, and the first of July, 1818, the weather was excessively hot and dry: no rain had fallen in a long time, and the thermometer in the shade was as high as 92 or 93 degrees. In the course of those three days, thirteen persons were admitted into the New-York Hospital with apoplexy; all of them labouring under more or less strongly-marked symptoms of that disease, said to have been occasioned by imprudently drinking large quantities of cold water. They were generally common labourers, foreigners, who had very recently arrived in this country, some of them having landed only a few days before at Amboy. It was stated that they had been at work exposed to the sun all the morning, and that they had drank more than their usual quantity of ardent spirits; that after dinner they returned to their work, and, with the additional excitement produced by eating, felt an increasing thirst, which obliged them to go frequently to the pumps, and to drink very largely of cold water.

"The symptoms which occurred, (and in some of the patients there was an opportunity of noticing them from the commencement,) were a sudden pain in the stomach, followed by slight vomiting, giddiness of the head, and fainting; shortly after, deep sighing, and difficult breathing; a rattling of the throat, with a secretion of a thick glairy mucus; the face was suffused with blood, and of a livid colour; the pulse was strong and full; the skin very hot and dry. In a short time after, coma supervened, and in some instances complete apoplexy, marked by stertorous breathing and loss of all voluntary motion: in most of them, tympanites occurred to a very great degree.

"The mode of treatment that seemed best adapted to this form of disease, was very large and repeated bleedings, stimulating enemata, and rubbing the whole surface of the body with aqua ammoniæ. This last application had a decidedly good effect in lessening the heat of the body and the comatose state of the patient, sometimes rousing him from an apparently fatal torpor. When the apoplectic symptoms had not yet supervened, laudanum, æther, and other stimulants, appeared to be beneficial; but their good effects were only momentary, for in a very short time afterwards apoplectic symptoms occurred. Several of the patients were bled to sixty, and one of them to eighty ounces, in the space of two hours. The recovery of this last patient was more complete and rapid than that of any of the others.

"As soon as blood was copiously withdrawn, the stertorous breathing ceased, and the senses were restored: immediately afterwards, a drink of cold water was asked for by the patient, and, when granted, was oftentimes instantly succeeded by vomiting; and the effect of it was highly beneficial, relieving the distended state of the stomach, and producing a general salutary impression. In some of the patients, symptoms of apoplexy returned after the second bleeding; and, in two cases, it was necessary to repeat the bleeding four times before the patients were completely relieved. On the next day, the breathing was remarked to be laborious, and in one instance it was with great difficulty that the patient could speak more than a few words at a time: he complained also of great pain and soreness of the right side; which, however, were soon relieved by the application of a blister to the affected part. Two of the thirteen patients died; one of whom, on examination, presented the following appearances:—The vessels of the brain extremely turgid; about five ounces of extravasated blood on the surface of the dura mater; some at the base of the brain; and the ventricles completely filled with serum; the lungs engorged, and of an appearance similar to the lungs of persons who have died of peripneumony; the heart red, and the pericardium much distended with a bloody-looking fluid; the stomach perfectly natural and quite empty.

"In all these cases, the action of the heart and arteries was greatly increased: in some of them it was excessively great, and the heart continued to palpitate very violently, even after the circulation at the wrist had been much reduced by copious bleeding. The heat of the body was likewise remarkably great, and in some instances continued for a long time after bleeding."

Those cases were popularly attributed to the drinking of cold water whilst the bodies of the patients were much heated; but Dr. Watts, from a comparison with the histories given by Drs. Rush, Jackson, and Currie, of the effects of that agent under such circumstances, doubts of the correctness of this opinion: he rather considers them to have arisen from the influence of the ardent sun. The author, after this, adduces some remarks on the supposed influence of cold water taken into the stomach whilst the body is inordinately hot, of general application. He thinks that the ill consequences of it may be attributed to the quantity taken distending the stomach in an enormous degree, in the cases of accidents from it on record, rather than to its quality with respect to temperature; since water in moderate quantities from the coldest springs, and even ice, are commonly taken under such a state of the system, without injurious effects. In his physiological explanation of the effects of this agent, when it has really been deleterious, his notions nearly coincide with those of Dr. Currie.

*Reflections on the Means of Recovery after Submersion, directed by the Humane Society of New-York. Addressed to the President and Directors.* By A. H. Stevens, m.d.

The observations made in this paper are chiefly of local interest alone. Dr. Stevens however objects, with propriety, to the measures dictated by the Humane Society of New-York for the recovery of persons after submersion; especially the use of injections of tobacco-smoke into the intestines. We believe this is still sometimes employed in England; but those who have witnessed the use of it for strangulated hernia, cannot doubt but that it must be deleterious. Dr. Stevens also reprobates the almost exclusive attention inculcated respecting the application of heat to the body, especially when children are the patients. He argues for the great importance of measures adapted immediately to produce respiration; but we cannot approve the method for effecting this by "rolling the patient on a cask," when medical assistance is not at hand: the people could readily be taught a more effectual, as well as a less dangerous, means for producing alternate dilation and contraction of the thorax.

*Cases of Fungus Hæmatodes of the Eye.* By A. H. Stevens, m.d.

These cases do not possess sufficient interest to detain us on the present occasion: they are, however, of value as additions to the histories we previously possessed of the same species of disease, but are not of sufficient novelty to induce us to transcribe them.

*A Case of circular Callous Ulcer at the Bottom of the Foot.* By Valentine Mott, m.d.

We discern nothing in this case calculated to be of general application, or particularly interesting except to its patient.

*A Singular Case of Disease of the Heart.* Communicated by Dr. Peter C. Tappen.

An abstract of this case was given in the last Number of our Journal.

*A Case of Hepatitis.* By John Watts, jun. m.d.

This affection occurred in a man, 30 years of age, and terminated in suppuration. The subsequent is the most interesting part of the history. The purulent matter was evacuated by vomiting, and for some time, to the amount of a pint ("of purulent and bilious-looking matter") in the twenty-four hours. After the rupture of the abscess, a slightly tonic method of treatment was adopted; but the patient getting worse, spitting more matter, and having hectic fever, cough, and increased pain in the region of the liver, it was changed for the antiphlogistic plan: tartarized antimony in nauseating doses; occasional loss of blood; a seton in the side; digitalis; and, at length, a light course of mercury. The patient finally recovered.

*A Case of Apoplexy.* By John Watts, jun. M.D.

The patient, a man aged 32 years, died five days after the attack of apoplexy. Copious and repeated abstraction of blood had been employed with temporary relief. On examination after death, "a large quantity of extravasated blood was found on the surface of the dura mater; the brain and its membranes were quite turgid; a considerable effusion was found in the left ventricle, and at the base of the brain. The inner table of the skull was affected by caries in different parts: about the centre of the left parietal bone, there was a caries of the size nearly of a sixpence, at which place a blood-vessel was eroded; accounting for the extravasation, and the singularity of the case."

*A Case of Chorea Sancti Viti.* By John Watts, jun. M.D.

This occurred in a gilder, whose occupation exposed him to the fumes of mercury. He had been for some length of time subject to convulsive muscular movements, and was affected with a slight degree of ptyalism. On his admission into the hospital, "his pulse being strong, full, and rather tense, sixteen ounces of blood were taken from his arm, and an active cathartic administered, which operated freely." The next day he was much better, and had not been disturbed the preceding night. He was directed to use the warm-bath, to have his body rubbed frequently with spirit of turpentine, and to take occasionally some cathartic medicine. Under this plan he continued to improve, until the 6th of February, when, in consequence, it is supposed, of being for some time exposed to cold, and getting his feet wet, his complaints returned with increased severity; the tremor and spasmodic affection of his body and limbs being so violent as to render it necessary, at times, to hold him on his bed: his pulse at this time was small, the perspiration profuse, and his strength much exhausted by the violence of the spasmodic paroxysms. He was put upon the use of wine, bark, and generous diet, and continued them for several days, but derived no benefit: the tincture of castor was then freely administered, and flannel bandages were very firmly applied to each of the extremities. The good effects of this last remedy, in controlling the convulsive affection of the muscles, was very apparent; for symptoms of chorea always returned when they were removed. Some power of voluntary motion was soon acquired, and in the course of a week he was able to walk. After taking laxative medicines, ammoniated tincture of valerian, and muriated tincture of iron, for some time, he was discharged cured.

*A Case of Intermittent Fever.* By John Watts, jun. M.D.

A well-marked case of quotidian intermittent fever, accompanied with cough and pain in the side, treated in the first instance by calomel and cinchona, but "without any effect on the fever, except rendering it apparently more violent. The cold fit was uniformly very long and severe, followed by inordinate arterial excitement, and severe pain in the head, back, and breast; the paroxysms terminating in profuse perspiration, and leaving the patient extremely feeble, with a slow and weak pulse." He was then repeatedly bled; calomel, tartarized antimony, and digitalis exhibited internally; and vesicatories were applied to his thorax and ankles. The type of the fever, in five days after this, changed to the tertian; and in a fortnight the patient was convalescent.

See the *Exposition of the Doctrines of* M. Broussais, in the last volume of this Journal, for some reflections on this subject.

This volume concludes with

*A Case of Brachial Aneurism, in which the Ligature was removed from the Artery Fifty-and-a-half Hours after its application.*

This case will engage our attention when we encounter it in the Transactions of the London Medico-Chirurgical Society, to which it has been related.

The great extent of our observations on the case of ligature of the arteria innominata, has obliged us to pass over in a rapid manner some other parts of this volume, which are perhaps the most valuable portion of its contents: we mean the papers by Dr. Watts, which afford an excellent specimen of the beneficial application of good and comprehensive physiological principles in the practice of medicine as an art. We look forward with anxiety for the continuation of a work containing the results of the labours of this physician.

---

*A Memoir on Inflammation of the Veins.* By G. Breschet, M.D. Prosector to the Faculty of Medicine of Paris, &c.

There are but few diseases that have been less understood, until within a late period, than that which forms the subject of this memoir. Observations respecting it may be found in the works of Areteus, Paré, Dionis, Platner, Boerhaave, Van Swieten, and Morgagni; but the nature of the affection was not determined until it was demonstrated by John Hunter. Since then, various instances of its occurrence have been published, a general reference to which was given by us on a former occasion, when considering the paper on this subject by Mr. Carmichael in the Transactions of the Association of the Dublin College of Physicians. The cases above alluded to being widely dispersed in medical literature, many of them have hitherto been unknown to the generality of surgeons; though, from the nature of the disease, it is of the highest importance that every one should possess all the information that can be furnished respecting it by historical records. To collect those cases, to arrange them in appropriate order, and to deduce from them some general conclusions, has been the object of M. Breschet in the memoir under consideration; and it is with much pleasure that we are enabled to offer to our readers the results of the labours of this intelligent and experienced surgeon on so interesting a subject.

We shall, in passing through this memoir, give a reference to the observations which may be supposed to be generally known to English surgeons; an abstract of those which have not hitherto been published in our language, and of those proper to M. Breschet; and conclude with the pathological reflections of this surgeon, and his remarks on the mode of treatment best adapted for their relief.

§ I. *Inflammation of veins caused by phlebotomy.*—In this section, Dr. Breschet adduces the cases related by Hunter, Shirwen, Mr. Abernethy, Mr. Charles Bell, and Mr. Travers; and details three instances of it which occurred to his own observation. The first patient, a man 22 years of age, who, having been bled in the arm several times for epilepsy, suffered after the last pain about the place of the incision; which was followed the next day by redness, tension, and swelling, extending in a few days from the shoulder to a little below the elbow. The pulse was weak and frequent; fever came on, with prostration of strength; very severe pain in the left side of the chest, with difficulty of respiration; which, after a few days, terminated in death. Examination of the parts then shewed the cephalic vein to be full of pus throughout its whole extent. The superficial radial vein contained also pus, until about two inches from its origin.

A woman, aged 20 years, was, on the 20th of July, bled in the left foot, for a vomiting of blood attributed to derangement in menstruation. She soon began to suffer very severe pain about the place of the incision, the edges of which became hard and everted. Swelling gradually extended to the superior part of the limb, accompanied with pain and tumefaction of the inguinal glands, fever, and restlessness. These accidents continued until the 23d, when they began to subside under the use of fomentations, cataplasms, rest, and laxative medicines. The saphena vein was then discovered, hard, tense, and increased in bulk, from about two fingers'-breadth below the ankle to the height of the origin of the tendon of Achilles. Pressure directed towards the opening, not yet cicatrized, produced an evacuation of a small quantity of thick pus, streaked with red. On the 26th, the pains and the suppuration had ceased, and the wound had healed. The vein, although diminished in diameter, still preserved its hardness, when the patient left the hospital on the 5th of August.

A man, 34 years of age, was bled three times within fifteen days from the right arm, for vague pains in the limbs of a year's standing: he was soon afterwards bled also in the left arm. On this occasion, symptoms of inflammation of the median cephalic vein ensued. "This case terminated favourably. On the sixteenth day from the accident, the vein had the appearance of a small cord, much less in diameter than the vein of the opposite side, evidently not admitting of the transmission of blood."

§ II. *On inflammation of veins produced by the ligature of them,*

*and by the excision of varices.*—"We know," says Mr. Breschet, "that Hippocrates punctured varices; that Ætius, Paulus Ægineta, Avicenna, and Albucasis, have described a method for the excision of them; that this operation appears to have been practised by Fallopius and Severinus; that the two Fabricii comprised the varicous portion between two ligatures, and emptied it by incision; and that Ambrose Paré, Dionis, Petit, and others, opened varices to extract the coagula from them, and afterwards approximated the sides of the vessels by compression. Nevertheless, a great number of facts attest, that the ligature and the excision of varices may induce serious accidents, of which death has been a frequent consequence."

M. Breschet then notices the results of the operation of tying the saphena major, practised a few years ago in St. George's Hospital; and the results of a similar measure related by Mr. Travers in his Surgical Essays.

M. Breschet has not noticed the important improvement in the mode of performing this operation, effected by Mr. Brodie, which has been attended with such advantageous results in his practice, and in that of Mr. Carmichael.

§ III. *Of inflammation of the veins after amputation.*—An abstract of the observations of Hunter and Mr. Travers, constitutes this section.

§ IV. *On inflammation of the veins consequent on the ligature of the umbilical cord.*—"Meckel," says M. Breschet, "has witnessed in an infant, a short time after its birth, vomitings, colics, diarrhœa, icterus, fever, and nervous affections: death took place on the tenth day. The peritoneum was inflamed and covered with a false membrane, and there was effusion of pus into its cavity. The branches of the vena porta, and especially those of the umbilical vein, were very tumid, and their parietes much thickened; those of the umbilical vein, and its first branches in the liver, were of the thickness of one or two lines, and were covered with a firmly-adherent spurious membrane. Osiander has published an analogous fact: an infant was attacked with erysipelas, that soon spread all over the body; and it died on the third day from its birth. The intestines and the liver showed signs of inflammation on their external surfaces; they were covered with false membranes and purulent matter. The umbilical vein, from the navel to the liver, contained pus of a yellow colour. Meckel has also furnished us with the history of an infant that was born with a strangulated inguinal hernia, consequent on compression of the abdomen during its passage from the uterus. The hernia could not be reduced before the third day; severe colics remained, accompanied with tympanitic distension of the abdomen, icterus, and vomitings. Death took place on the seventh day. On opening the body, all the disorganization consequent on peritonitis was discovered; the parietes of the umbilical vein were much inflamed, and covered with pus; and there were spots of ulceration on its internal surface."

The analogous observations published by Dr. Colles,* of Dublin,

---

* *Dublin Hospital Reports*, vol. i. See *London Medical and Physical Journal*, vol. xxxix. for an abstract of these observations.

who has witnessed *trismus* as a frequent attendant on this disease, have escaped the notice of M. Breschet.

§ V. *On inflammation of the crural veins after parturition.*—Several cases of this affection have been published by Meckel.* "A woman died a few days after parturition. A short time after her delivery, she was attacked with fever, and darting pains in the cavity of the abdomen and in the pelvis, which after a time disappeared; but, at the end of about three weeks, uneasiness in the region of the liver and in the left hip, and intolerable pain in the left thigh, supervened. On examining the dead body, the abdominal cavity was found filled with purulent matter; the liver was very voluminous, the lungs healthy. The crural vessels and nerves were surrounded with puriform matter. The crural vein, from its origin to the knee, was of the thickness and the firmness of the artery; it was filled with pus and blood. Its tunics cracked under the scissars; its internal membrane, well washed, was more spongy than in the ordinary state, and covered with a very distinct false membrane, which might be detached from it in slips. Its valves were in part corroded, ragged; and in part thickened, tumified, and of a deep-red colour."

The analogous observations of Mr. Travers, Mr. Wilson, and Dr. Clarke, are next cited; and this section concludes with a remark, that "M. Chaussier has frequently seen, in women who died after parturition, the abdominal veins full of sanious pus; and M. Ribes has observed, in a woman who died of peritonitis, at the same epoch, the same veins gorged with purulent sanies."

We may here remark, that, as no mention is made of the state of the vessels themselves in the latter cases, it is not satisfactorily shown that they were instances of *phlebitis;* the "sanious pus" may have been taken up by them from extraneous sources.

§ VI. *On inflammation of the veins of the uterus and the ovaries after abortion.*—"A woman," says M. Breschet, "having aborted in the seventh month of gestation, the placenta being necessarily extracted by art, was attacked with fever, a miliary eruption, and pains in the loins and in the pelvis. Purulent matter flowed from the genital organs and from the rectum. After death, there was found, between the posterior paries of the uterus and the vagina, an abscess which opened into the rectum. The veins of the uterus and the ovaries were inflamed, their parietes thickened, their cavities ulcerated and filled with pus. The whole of the right renal vein, and th part of the vena cava that receives it, were inflamed, red, tumefied, and filled with a coagulum of blood, which in its middle contained some puriform matter."†

§ VII. *On inflammation of the veins by their direct communication, or their contact, with other diseased structures.*—M. Fizeau has published a case‡ of suppuration in the hepatic veins, connected with organic affection of the biliary canals; in which M. Breschet is disposed to attribute the inflammation producing the former affection to

---

* *Bibliothèque Medicale*, tome xvi.

† *Ibid.* tome xvi.

‡ *Ibid.* tome xxxviii.

the latter cause. M. Breschet next adverts to the observations on this subject in the essay of Mr. Travers, and then relates a case that occurred to M. Patissier: "In a subject who died of tetanus, the internal membrane of the auricles and ventricles of the heart, especially of the right side, were found of an intense red colour, chiefly at the basis of the mitral and tricuspid valves: the internal membrane of the aorta, the common trunks of the carotids, the subclavian arteries, the inferior vena cava, the jugular veins, and the pulmonary artery, presented red appearances that diminished in proportion as they diverged from the heart, which was not removed either by repeated washing, or even on scraping the membrane with a scalpel."

M. Ribes has often met with inflammation of the veins in erysipelas. He says, that, if this disease induces suppuration, which is very rare, the parietes of the veins are red, especially the internal membrane; their thickness is also increased, and their cavity filled with pus. In the case of gangrene, they are black, easily torn, and contain sanious matter. If the gangrene has not proceeded to the last stage, the parietes of the veins are a little dilated and very thick; their internal membrane, at the circumference and in the neighbourhood of the focus of the disease, are red, and visibly inflamed: they contain pus or sanies. M. Ribes has sometimes met with this sanies to the extent of several inches from the focus of the malady.*

§ VIII. *On inflammation of veins produced by metastasis.*—A boy, 14 or 15 years of age, was affected with an eruption that had been considered to be the itch, which soon disappeared on the use of some ointment. He was almost immediately attacked with violent and continued pain in the right lower extremity, which became the seat of serous effusion. When he entered the hospital, he had all the symptoms of what is commonly termed nervous continued fever: he died after about three weeks. The vena cava was blackish in colour in some parts, whitish in others, was easily torn, and contained a white medullary matter, not coherent, which increased in quantity as it extended towards the iliac trunks, before the bifurcation of which the vessel was dilated and completely obstructed by the matter just described. The trunk of the left iliac veins was also obstructed by it, and the branches of it, although much dilated, entirely empty. In place of the iliac trunk of the right side, there was a sort of ligamentous canal, with thick parietes, and extremely small calibre, which terminated in a vast abscess formed in the cellular tissue surrounding the hypogastric and iliac vessels. Notwithstanding the most careful research, not the least vestige of the right crural vein could be found: its course was occupied by a train of pus, which extended to the ham. The veins of the right leg were contracted, and filled with solidified fibrine.

§ IX. *On inflammation of veins from chemical or mechanical causes, the presence of worms, &c.*—Treutter, Fabricius, and Rudolphi, describe worms which have been found in veins. Pus absorbed from ulcers of a malignant character, hospital gangrene, &c. some cases of

* *Memoires de la Societe Medicale d'Emulation,* tome viii.

caries of the bones, have been attended with inflammation of the veins. Sasse produced it in dogs, by injecting solution of opium, and tincture of euphorbium and cantharides, either into the jugular veins or into the cavity of the abdomen.

We defer the remainder of this memoir to the next Number, which will comprise the general conclusions of M. Breschet on the foregoing varieties of disease.

---

*Cases of Somnambulism and Epilepsy.* By Dr. Martinet.

[From the *Bibliotheque Medicale,* Fev. 1819.]

"—— Massy, aged 25 years, a saddler, born of parents who have not evinced any thing remarkable with respect to the functions of the brain, experienced, when thirteen years of age, violent terror from a man suddenly and forcibly stopping him in the street: three days afterwards, having suffered some vexation, he was seized with convulsions and subsequent insensibility, which continued three-quarters of an hour. A short time afterwards, a second attack manifested itself; and, during the course of this month, the paroxysms recurred every day, increasing each time in length of duration: in other respects, his health was good, except that the least moral opposition induced an attack of his malady. The use of his intellectual faculties returned but slowly after them. For three months he used a cold bath, and in the third had not suffered a single attack. They ceased completely until the nineteenth year of his age, when he became a somnambulist, working during the night at his trade as a saddler, getting out on the roof of the house, going out to walk, and occupying himself in a thousand various ways. Soon after this, the fits of epilepsy re-appeared, recurring every five or six days, increasing in duration, and commencing, from then only, with a sensation of heat, which, from the epigastrium, rapidly extended to the head, and induced insensibility. These affections disappeared after the use of cold affusions for about a month. On returning home to his parents, new vexations induced him to attempt to destroy himself; and he, indeed, hung himself by the feet. Having been found in this state, he was bled several times, and at length recovered the use of his senses. From this time until the age of twenty-one, he had no attacks of epilepsy; but at that period they again appeared, but without being announced, as formerly, by a sensation of heat at the epigastrium: their invasion was much more sudden, and, after their cessation, the toes remained contracted for at least two hours. Cold affusions again removed them, and he was a year without a fit. At the age of twenty-two, they recommenced, occurring after intervals of a few months. When twenty-three years of age, at the instant of the accession of a fit, the pole of a carriage fell on his head: the commotion which this produced, rendered blood-letting necessary. He entered into the army, and was three months without a paroxysm. (He then got bled from time to time.) The following year, the twenty-fifth of his age, he was much astonished to find himself, one night, on the roof of the house, wet with rain: the impression which he thence conceived, produced a short time afterwards an attack of epilepsy, which was followed by contraction of his fingers and toes.

"About this period he entered the Hôtel-Dieu. Leeches were repeatedly applied along the vertebral column, and he was also bled from the arm several times. The tetanic attack, which, for some time past, preceded and accompanied each fit, diminished considerably; and after about six weeks he was able to resume his occupation.

"J. Béatrix, 22 years of age, born of parents who have never manifested any particular cerebral disorder, experienced, for the first time, after a fit of terror, complete loss of sensibility.

"When fourteen years of age, being apprenticed to a watch-maker, he sought for a shoe-brush one day to clean a watch with it. His master, astonished at so strange an action, when his replies to questions were perfectly appropriate, gave him, in a moment of anger, a blow on the head: the young Béatrix again fell into a state of insensibility. When this fit had terminated, which lasted about five minutes, he had no recollection of what had passed: it was from his comrades only that he learned, both what he had suffered, and the action by which it had been provoked.

"Three months after that occurrence, he had a new fit of somnambulism, which set in with a sensation of heat, extending rapidly from the epigastrium to the head, followed by confusion of thought and complete insensibility. From that time, the paroxysms of somnambulism were renewed about every fifteen days. At the time of their duration, the eyes were fixed and open; and Béatrix then employed himself at all the affairs that formed the subject of his occupations in his waking hours, and was very much surprised to find his work in a different state from that in which it had been before the commencement of his sleep: sometimes however he recalled to mind the different objects that had struck him very forcibly. Between the age of twenty-one and twenty-two, an attack of another nature manifested itself: having experienced a violent moral impression, he suddenly fell to the ground, with loss of sensibility, general convulsions, retraction of the limbs, and frothing at the mouth. This state continued for two hours. Since that time, the accessions of epilepsy and somnambulism have happened very frequently, notwithstanding the use of cold affusions, that were employed with the view to their alleviation."

